# Comparative evaluation of phenyl isothiocyanate derivatization and “dilute-and-shoot” methods for HPLC–MS/MS-based targeted metabolomics analysis of amine-containing metabolites in plasma samples

**DOI:** 10.1007/s00216-025-06079-5

**Published:** 2025-08-30

**Authors:** Kangkang Xu, Markus Aigensberger, Franz Berthiller, Heidi E. Schwartz-Zimmermann

**Affiliations:** 1 Institute of Bioanalytics and Agro-Metabolomics, Department of Agricultural Sciences, BOKU University, Konrad-Lorenz-Straße 20, 3430 Tulln, Austria; 2 Christian Doppler Laboratory for Innovative Gut Health Concepts of Livestock, 1210 Vienna, Austria

**Keywords:** Derivatization, High-performance liquid chromatography, Mass spectrometry, Amino acids, Biogenic amines, Method comparison

## Abstract

Metabolomics, the study of small molecule metabolites in biological systems, is essential for disease diagnosis and biomarker discovery. A key consideration in developing targeted metabolomics methods using HPLC-MS/MS for human or animal plasma is whether to employ derivatization of amino acids, amino acid-related compounds, and biogenic amines. Derivatization with phenyl isothiocyanate (PITC) enhances ionization and LC-separation, but complicates sample preparation and introduces potential errors. This study focuses on (1) the validation of a PITC derivatization method employing reversed-phase (RP) HPLC-MS/MS analysis and (2) a comparison of two analytical approaches for targeted metabolomics analysis of animal and human plasma: the PITC derivatization-based RP-LC-MS/MS method and a “dilute-and-shoot” approach using hydrophilic interaction chromatography (HILIC)-MS/MS and RP-LC-MS/MS analysis. The derivatization method was validated for porcine plasma, assessing limits of detection, lower limits of quantification (LLOQs), linearity, repeatability, recovery, and trueness. Derivatization reduced LLOQs for derivatized compounds in pure solvent solutions but, due to higher dilution factors, resulted in similar LLOQs for derivatized compounds and higher LLOQs for non-derivatized compounds in plasma compared to the “dilute-and-shoot” method. Derivatization improved chromatographic separation of isomers and reduced carryover but introduced challenges such as matrix effects, coelution with impurities, and calibration issues. The “dilute-and-shoot” method performed better for non-derivatized compounds and was less error-prone. Both methods were applied to plasma from various species, demonstrating comparable concentrations for most metabolites. The results also emphasize the importance of using different approaches for cross-validation. Above all, this study highlights the strengths and limitations of both the derivatization method and the “dilute-and-shoot” approach, providing guidance for their application in targeted metabolomics.

**Graphical Abstract:**

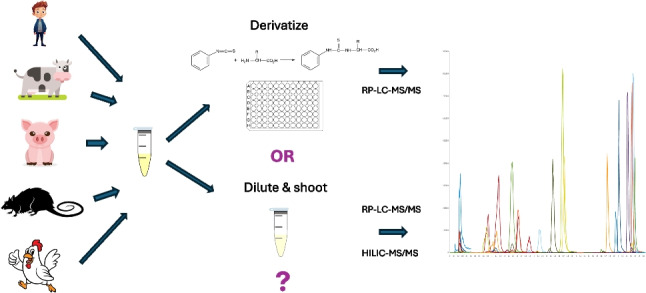

**Supplementary Information:**

The online version contains supplementary material available at 10.1007/s00216-025-06079-5.

## Introduction

Metabolomics, the study of small molecule metabolites in a biological system, is a powerful tool for investigating the influence of endogenous or exogenous factors on that system. Applications include early disease diagnosis, biomarker discovery, nutritional evaluation, and overall health assessment [1–3]. The metabolome is chemically diverse, consisting of metabolites from various chemical classes that exhibit significantly different physicochemical properties and exist across a broad concentration range within biological systems [4]. This circumstance complicates the development of analytical methods for quantitative metabolomics.

Nevertheless, a lot of efforts have been put into the establishment of targeted liquid chromatography tandem mass spectrometry (LC-MS/MS) based methods for sensitive quantitative metabolomics in the last decade, e.g., [5–15]. These methods differ, among others, in the number of analytes and compound classes covered, in the selected biological matrices, in the sample preparation and analysis method itself, as well as in the extent of method validation. The compound classes covered in most methods for human and animal metabolomics are amino acids and amino acid-related compounds, biogenic amines, bile acids, organic acids, fatty acids, nucleotides, sugars and derivatives, acyl carnitines, steroids, and other lipids. Li et al. [7] managed to develop a hydrophilic interaction chromatography (HILIC) method capturing all of the above mentioned compound classes, albeit with some limitations like poor peak shapes and coelution of certain analytes. As no single LC method can cover the entire metabolome, most research groups developed a set of method combinations for their specific analytes. These include combinations of reversed-phase (RP) chromatography methods with different stationary phases, mobile phases and gradients [5, 11], combinations of RP and HILIC [6, 8, 16] as well as combinations of RP and anion exchange chromatography (AIEX) [15, 17]. Since achieving effective separation of highly polar and partially (zwitter-)ionic compounds, such as amino acids, biogenic amines, and organic acids, on traditional RP C18 phases is challenging, several research groups have resorted to derivatization of these compounds [11, 17]. Derivatization with phenyl isothiocyanate (PITC) followed by RP-LC-MS/MS analysis is also widely used in commercial kits for targeted metabolomics (e.g., AbsoluteIDQ® p180 kit, MxP® Quant 500 kit, both Biocrates, Innsbruck, Austria), and has the additional benefit of enhancing compound ionization during mass spectrometric analysis and detection. However, due to multiple pipetting and evaporation steps, derivatization is more error-prone than simple dilution or protein precipitation and consequently significantly complicates sample preparation.

While PITC derivatization of plasma metabolites followed by RP-LC-MS/MS analysis is a widely established technique in human and animal metabolomics, there are limited validation data available. Zheng et al. [11] validated a PITC derivatization assay for the analysis of amine-containing compounds in human urine, but no validation studies using PITC derivatization with plasma as a matrix have been published to date. The first aim of our present work is to validate our previously published and slightly modified PITC derivatization-based RP-LC-MS/MS method for quantitative determination of amino acids, amino acid-related compounds, and biogenic amines in porcine plasma [17] with respect to limits of detection (LODs) and quantification (LOQs), linearity, repeatability, apparent recovery (RA), carryover, and trueness. Our second aim is to compare this method with our recently published set of “dilute-and-shoot” based RP-LC-MS/MS and HILIC-MS/MS methods for quantification of 235 compounds from 17 classes [16]. Special emphasis will be put on comparison of LODs and LOQs, linear range, repeatability, RAs, and metabolite concentrations obtained in plasma samples from pig, human, rat, bovine, and chicken that will be analyzed with and without derivatization. This comparison will elucidate the strengths and limitations of both methods and help in the decision whether to (still) employ derivatization or to solely rely on “dilute-and-shoot” methods.

## Materials and methods

### Reagents and standards

Methanol (MeOH, LC-MS grade) and ethanol (p.a.) were purchased from Merck (Darmstadt, Germany). Acetonitrile (ACN, LC-MS grade) was procured from VWR International GmbH (Vienna, Austria); isopropanol (IPA, LC-MS grade) and formic acid (MS grade) were from Honeywell (Vienna, Austria). PITC (99% for protein sequencing), anhydrous pyridine (99.8%) as well as ammonium acetate (for MS) were obtained from Sigma-Aldrich (Vienna, Austria). LC-MS grade water was produced using a Sartorius Arium® Pro water purification system (Sartorius, Göttingen, Germany) and used in all experiments.

**Fig. 1:**
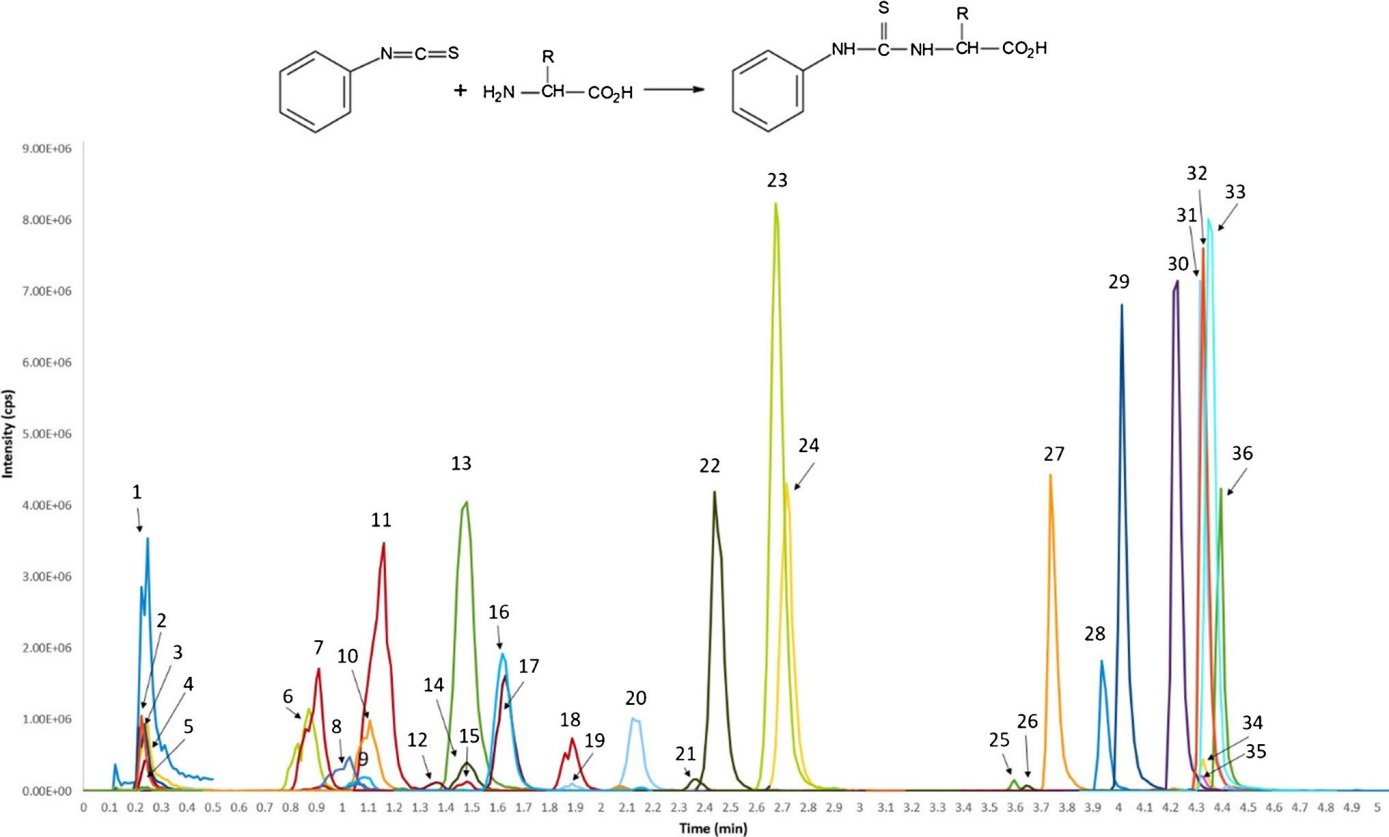
LC-MS/MS chromatogram of selected amino acids, amino acid-related compounds and biogenic amines derivatized with PITC in pig plasma. 1, betaine; 2, choline; 3, carnitine; 4, creatine (non-derivatized); 5, trigonelline; 6, histidine; 7, taurine; 8, anserine; 9, 3-methylhistidine; 10, trans-4-hydroxyproline; 11, arginine; 12, asparagine; 13, glutamine; 14, pyroglutamic acid; 15, ethanolamine; 16, serine; 17, glycine; 18, citrulline; 19, methionine sulfoxide; 20, glutamic acid; 21, dimethylamine; 22, threonine; 23, proline; 24, alanine; 25, serotonin; 26, 3-indolepropionic acid; 27, tyrosine; 28, methionine; 29, valine; 30, ornithine; 31, tryptophan; 32, creatine (derivatized); 33, lysine (original peak area/3); 34, isoleucine; 35, leucine; 36, phenylalanine. The reaction at the top depicts the derivatization of amino acids with PITC (C1 = CC = C(C = C1)N = C = S)

A list providing CAS numbers, sum formulas, molecular masses, and providers of the metabolites investigated in this study is given in Table S1. Stock solutions of 1000 mg/L for single compounds were prepared by dissolving reference compounds in ACN/water (50/50, *v*/*v*) unless stated otherwise in Table S1, and stored at - 80 °C. The internal standards, including ^13^C-putrescine (^13^C_4_, 98%) and the deuterated cell-free amino acid mix (20 amino acids, 98%, hydrogen atoms replaced by deuterium only in the “R” part of the amino acids as shown in Fig. 1) containing 20 amino acids at different ratios, were purchased from Eurisotop (Tewksbury, MA, USA). Internal standard stock solutions containing 1000 mg/L of ^13^C-putrescine and 1000 mg/L of the deuterated amino acid mix were prepared in ACN/water (50/50, *v*/*v*) and stored at - 80 °C.

### Plasma samples

Porcine plasma was sourced from a pig herd of the pig facility (Vetfarm) of the University of Veterinary Medicine Vienna, and a pooled quality control (QC) sample was prepared as detailed in [16]. Lyophilized plasma from rat, bovine, human, and chicken was obtained from Sigma-Aldrich (Vienna, Austria) and reconstituted in the indicated volume of purified water. Additionally, frozen human plasma stemming from the National Institute of Standards and Technology (NIST, Portland, USA) was purchased to investigate the method trueness.

### Sample preparation

As outlined in our previous work [17], sample preparation for the derivatization method was performed according to a modified protocol based on Biocrates’ MxP® Quant 500 kit (Biocrates, Innsbruck, Austria). 25 µL aliquots of internal standard solution (11 mg/L of ^13^C-putrescine, 989 mg/L of deuterated amino acid mix) were pipetted into 96-well plates and different volumes of mixed calibration solutions or 10 µL of plasma were added. After evaporation to dryness in a Centrivap vacuum concentrator (Labconco, Kansas City, MO, USA), 50 µL of derivatization reagent (ethanol/water/pyridine/PITC 31.7/31.7/31.7/5.0, *v*/*v*/*v*/*v*) was added, the plate was covered, shaken for 20 s, and compounds with amino groups were derivatized in the dark at ambient temperature for 1 h. Subsequently, the derivatization reagent was evaporated in the Centrivap concentrator and the analytes were extracted into 300 µL of MeOH containing 4.9 mM ammonium acetate by shaking for 30 min. The solutions were measured directly (overall plasma dilution factor 30) and after 1 + 49 (*v* + *v*) dilution (overall plasma dilution factor 1500) with MeOH by LC-MS/MS.

Sample preparation of the “dilute-and-shoot” approach consisted of protein precipitation by mixing 200 µL of plasma with 800 µL of cold IPA, centrifugation, and further 1 + 19 (*v* + *v*) dilution of supernatants with IPA/water (80/20, *v*/*v*) as described in [16]. Both dilutions (1:5 and 1:100 dilution in total) were subsequently measured by LC-MS/MS in HILIC and RP mode.

### LC-MS/MS measurement

For both approaches (derivatization and “dilute-and-shoot”), LC-MS/MS analysis was carried out on a 1290 Infinity II series UHPLC system coupled to a 6500 + triple quadrupole mass spectrometer equipped with a Turbo Spray IonDrive source (Sciex, Foster City, CA, USA). In the derivatization method, chromatographic separation was achieved at a temperature of 50 °C in gradient elution on a Kinetex C18 column (50 × 2.1 mm, 2.6 µm particle size, Phenomenex, Aschaffenburg, Germany) protected by a C18 SecurityGuard ULTRA pre-column (Phenomenex). Mobile phase A was water, mobile phase B was ACN (both containing 0.2% formic acid, *v*/*v*). The following gradient was used: 0.0–0.3 min: 5% B, 0.3–2.7 min: linear increase to 15% B, 2.7–4.0 min: linear increase to 40% B, 4.0–5.0 min: linear increase to 100% B, 5.0–6.0 min: isocratic washing period at 100% B, 6.1–7.5 min: re-equilibration at 5% B. The flow rate was 0.6 ml/min (except for the period from 5.2 to 6.1 min where it was increased to 0.7 ml/min) and the injection volume was 2 µL.

Mass spectrometric detection was carried out in scheduled selected reaction monitoring mode (sSRM) in positive ionization mode. The ion source parameters were: temperature 500 °C, ion spray voltage 5500 V, curtain gas 40 psi, ion source gas 1 60 psi, and ion source gas 2 70 psi. Derivatization state, compound specific sSRM parameters, analyte retention times, and ion ratios for unequivocal compound identification are listed in Table S2a. Sciex OS version 3.0.0.3339 was employed for instrument control and data evaluation.

Chromatographic separation and mass spectrometric detection of non-derivatized samples by the “dilute-and-shoot” approach has been described in detail in [16]. In short, HILIC separations were performed on a Kinetex HILIC column (150 × 2.1 mm, 2.6 µm, Phenomenex, Aschaffenburg, Germany), employing gradient elution with ACN/water mobile phases modified with 1 mM ammonia and 0.1% formic acid (*v*/*v*). RP chromatography was carried out on a Kinetex C18 column (150 × 2.1 mm, 2.6 µm) in gradient elution using mobile phases consisting of IPA/ACN/water that were equally modified with ammonia and formic acid. Both gradients are listed in Table S2b. Tandem mass spectrometric detection was achieved in sSRM mode with fast polarity switching [16]. Compound specific sSRM parameters, analyte retention times, and ion ratios are given in Table S2a, and ion source parameters are stated in Table S2b.

### Validation of the derivatization method

For determination of LODs, lower LOQs (LLOQs) and the linear range (expressed by the upper LOQ (ULOQ)), a mixed stock solution containing 18 mg/L of all analytes was prepared and serially diluted in factors of 10, with the lowest dilution containing 0.0018 mg/L. Different volumes (17–59 µL) of the prepared standard mixes were added to the 96-well plate and evaporated to dryness, resulting in calibration curves ranging between 0.0001 and 3 mg/L in measurement solution. LODs and LLOQs were defined as the concentrations corresponding to signal-to-noise (S/N) ratios of 3 and 10, respectively. Furthermore, the concentration at the LLOQ was calculated based on the established calibration curve and had to fall within 70% to 130% of the theoretical value. ULOQs were determined through visual inspection of the calibration curves, and the back-calculated ULOQ was required to be between 95 and 105% of the actual concentration.

Apparent recoveries (RAs) for plasma samples were determined by standard addition. To this end, different volumes (10–60 µL) of a mixed stock solution containing between 0.08 and 75 mg/L of all analytes were spiked into the 96-well plate containing either only 25 µL of internal standard mix (solvent calibration curve) or 25 µL of internal standard mix and 10 µL of QC plasma (standard addition curve). For most compounds, the spiked concentrations corresponded to 0.5, 1.0, 1.5, 2.0, 2.5, and 3.0 times the previously determined analyte concentration in QC plasma. To avoid exceeding the ULOQ during validation, highly abundant compounds were spiked at levels corresponding to 0.17–1.0 times the analyte concentration in QC plasma. For compounds < LLOQ, a concentration of 0.150 mg/L in plasma was designated as level 1, and the other spiking levels (from 0.5 × level 1 to 3 × level 1) were calculated accordingly. Derivatization was then carried out as described above. Each solvent calibration curve and each standard addition curve was prepared in triplicate and measured, both concentrated and after 1 + 49 (*v* + *v*) dilution, by LC-MS/MS. RAs in plasma were primarily calculated by dividing the slope of the standard addition curve in the plasma extract by the slope of the preceding solvent calibration curve. For all compounds, regression lines were established by plotting peak areas against the spiked concentrations. For amino acids and putrescine that possess a dedicated internal standard, additional regression lines were obtained by plotting peak area ratios (analyte peak area divided by the peak area of the respective internal standard) against the spiked concentrations. The RAs from three replicates were then averaged. In cases where quadratic regression was required (primarily if the highest spiking levels surpassed the ULOQ), RAs were determined by subtracting the concentration in the unspiked sample from the concentration at the individual spiking levels (calculated based on the preceding quadratic calibration curve) and then dividing the difference by the spiked concentration at the respective level. The mean RA was calculated by averaging the RAs obtained from 6 levels derivatized and measured in triplicates (*n* = 18). For all compounds, RAs were determined in concentrated samples. For analytes quantified in diluted samples, RAs were also determined in diluted samples.

The repeatability was assessed by calculating the relative standard deviation (RSD) of the recoveries determined at 6 spiking levels in triplicates (*n* = 18). RAs were determined as described above, using preferably linear or, if required, quadratic calibration curves. Analyte carryover was determined by injecting a pure solvent blank after each 0.30 mg/L and 1.0 mg/L standard and calculating the peak area ratios (%) in the blanks compared to the standard solutions. For compounds with LOQs greater than 1.0 mg/L, the peak area of the highest validation level was compared with that of the blank measured directly afterwards. Trueness was evaluated by quantifying the analytes present in the certified reference plasma (NIST, Portland, US) and then comparing the results with the certified values.

Validation of the “dilute-and-shoot” approach was carried out very similarly as described for the derivatization method. A detailed description is given in [16].

### Quantification of analytes in animal plasma by the derivatization method

All plasma samples were derivatized in duplicate and measured at two dilutions (1:30 and 1:1500, *v:v*). Analytes in QC plasma, reference plasma, and plasma samples from different animal species were quantified based on 7-point pure solvent calibration curves covering a concentration range of up to three orders of magnitude and taking into account LLOQs, ULOQs, and the expected concentration range of each analyte. Calibration curves for compounds with dedicated internal standards (amino acids and putrescin) were established by plotting peak area ratios (analyte peak area divided by the peak area of the respective internal standard) against the concentrations. Concentrations of all other compounds were corrected using the previously determined RAs by dividing the concentrations obtained from the calibration curves by the respective RAs and multiplying by 100. Final concentrations in plasma were then calculated by applying the respective dilution factors.

### Comparison of the derivatization and “dilute-and-shoot” methods

The “dilute-and-shoot” method and its validation are described in detail in our recent publication [16]. For method comparison, the same plasma samples were measured by both the derivatization and the “dilute-and-shoot” method, and analytes were quantified on the basis of calibration curves established from the same stock solutions, considering the respective RAs.

## Results and discussion

### Derivatization reaction

Derivatization with PITC is frequently used for the analysis of amino acids, amino acid-related compounds, and biogenic amines, both in standalone analytical procedures and in commercially available assay kits, e.g., [17–19]. The underlying reaction is shown in Fig. 1. Of the 75 investigated compounds in our study, 18 were not derivatized, while the majority of 49 compounds were derivatized once, six compounds were derivatized at two positions of the molecule, and two compounds were partially derivatized (Table S2a). All 19 investigated proteinogenic amino acids (cysteine was unstable and therefore omitted) were derivatized, with lysine - featuring a second free amino group - being derivatized at both amino groups. The percentage of non-derivatized compounds was higher for amino acid-related compounds. Twelve amino acid-related compounds were not derivatized, and two compounds (creatine and pyroglutamic acid) occurred in both derivatized and non-derivatized form. In the case of *N*-acetyltyrosine, hippuric acid, nicotinuric acid, phenylacetylglycine, and arachidonoyl glycine, acylation of the amino group (resulting in formation of a secondary amide) prevented derivatization. Pyrrol-type nitrogen atoms as present in 3-indoleacetic acid and 3-indolepropionic acid were not derivatized, either. Similarly, quaternary ammonium compounds like betaine or stachydrine cannot be derivatized. Interestingly, although proline, containing a secondary amine structure, could be derivatized, *N*-methylalanine could not. Creatine occurred in both derivatized and non-derivatized forms, indicating an incomplete yield of the derivatization reaction. Derivatization took place on the amino group, which is part of a more complex guanidino group. Interestingly, guanidinoacetic acid, the biological precursor of creatine, was not derivatized. Another contradictory result was obtained for pyroglutamic acid, which, although containing a secondary amide group, occurred in derivatized and non-derivatized form. In contrast to the other derivatized compounds, sarcosine was not stable after derivatization, and derivatized sarcosine slowly degraded over the course of the measurement sequence.

Of the 20 investigated biogenic amines, six were not derivatized, ten were derivatized at one position, and four were derivatized at two or more positions. The non-derivatized compounds were either quaternary ammonium compounds (carnitine, choline, trimethylamine-*N*-oxide, trigonelline) or amides (benzamide, creatinine). The diamines cadaverine and putrescine were derivatized at two positions, whereas the triamine spermidine - possessing two primary and one secondary amines - was derivatized at two and three functional groups. Likewise, the tetraamine spermine occurred in 2x, 3x, and 4× derivatized form. For both spermine and spermidine, the double derivatized form was predominant and therefore used for identifying and quantifying both analytes. As pyridine was used as a reagent in the derivatization reaction, the biogenic amine 2-hydroxypyridine could not be determined by the derivatization method.

### Comparison of LC-MS/MS analysis by both methods

Derivatization with PITC enhances the retention of polar compounds containing primary or secondary amines, which are otherwise poorly retained in RP chromatography. For instance, as described in our previous article [12], only the amino acids phenylalanine and tryptophan, the amino acid-related compound nitrotyrosine, and the amines serotonin, kynurenine, 2-phenylethylamine, and thyroxine had retention factors > 1 in RP-HPLC using a C18 column. Derivatization increased the average retention factors of amino acids, amino acid-related compounds, and biogenic amines to 12.2, 9.1, and 14.8, respectively (Table S2a).

A chromatogram showing the major metabolites in pig plasma determined by the derivatization method is shown in Fig. 1. Chromatograms showing the major metabolites in non-derivatized pig plasma recorded by HILIC- and RP-LC-MS/MS are given in the Electronic Supplementary Figures S1 and S2. Notably, all compounds that were derivatized in this study showed retention factors > 3.9 when analyzed as native compounds in the employed HILIC method (Table S2a).

Similar to the “dilute-and-shoot” method [16], the isomeric amino acids leucine and isoleucine coeluted in the derivatization method. However, isoleucine can be distinguished due to a specific SRM transition to the fragment of *m/z* 69 that is not formed from leucine. The quantifier ion of leucine (*m/z* 43) has a much weaker intensity than the qualifier ion of *m/z* 132, but *m/z* 43 is formed predominantly from the parent ion of leucine. Glycine could not be reliably determined in the “dilute-and-shoot” method due to its small molecular mass and limited fragmentation. Derivatization yielded reliable quantifier and qualifier transitions for glycine. However, the first isotope peak of naturally occurring glycine (2.2% of ^12^C-glycine) interferes with the transition of the internal standard, in which only one hydrogen atom was replaced by one deuterium atom. The extent of this interference depends on the natural concentration of glycine and can be more than 10%. Hence, as discussed in the validation section, it is not recommended to use this particular internal standard mixture in the case of glycine. Another issue caused by the used internal standard mixture is an unidentified impurity almost completely coeluting with histidine that shares both quantifier and qualifier transitions of the amino acid, albeit with a different ion ratio. As the impurity gives rise to an intense peak of similar area as naturally occurring histidine in concentrated plasma samples, histidine cannot be reliably quantified in samples with internal standard.

Concerning amino acid-related compounds, 1-methylhistidine and 3-methylhistidine coeluted in the derivatization method, but were well separated in the HILIC “dilute-and-shoot” approach. However, both compounds have specific transitions, enabling correct quantification in the derivatization method as well. The isomers α-aminobutyric acid (AABA), β-aminobutyric acid (BABA) and γ-aminobutyric acid (GABA) were baseline-separated after derivatization, but their isomer *N*-methylalanine completely coeluted with the intrinsically cationic compound choline whose qualifier transition (*m/z* 104 → 58) was identical to the quantifier transition of *N*-methylalanine. As the ion ratio of *N*-methylalanine was 0.07, use of the specific qualifier transition would result in unacceptably high LLOQs (see Table S3). In the “dilute-and-shoot” method, GABA partly coeluted with AABA, but had specific SRM transitions. BABA coeluted with *N*-methylalanine, and both compounds slightly coeluted with AABA. However, BABA had a quantifier transition to *m/z* 44 that was only very small in the MS2 spectra of AABA and *N*-methylalanine. Likewise, the quantifier transition of AABA and N-methylalanine (*m/z* 104 → 58) was not visible for BABA. Choline, which is isobaric to the protonated molecular ions of AABA, BABA, GABA, and *N*-methylalanine, was baseline-separated from AABA and almost baseline-separated from BABA and *N*-methylalanine. The isomeric compounds alanine, β-alanine, and sarcosine were separated after derivatization, but coeluted in the “dilute-and-shoot” method. As no specific transitions were available, only a sum determination was possible in the “dilute-and-shoot” approach. Likewise, 3-hydroxyproline, cis-4-hydroxyproline, and trans-4-hydroxyproline were at least partially separated in derivatized form, whereas they completely coeluted in the “dilute-and-shoot” method. Similarly, *N*,*N*-dimethylarginine (ADMA) and *N*,*N*′-dimethylarginine (SDMA) were better separated in derivatized form, but also had specific quantifier transitions (*m/z* 203 → 158 for ADMA, *m/z* 203 → 172 for SDMA) in the “dilute-and-shoot” approach. In contrast, valine and norvaline completely coeluted after derivatization, whereas partial separation was achieved in the HILIC “dilute-and-shoot” method. The highly polar biogenic amine spermidine could neither be retained in RP chromatography on a C18 column, nor could it be eluted from the HILIC column due to too high retention [12]. In the derivatization method, it eluted late with a retention factor of 20.3, but it could still be quantitatively determined.

### Validation of the derivatization method and comparison to the “dilute-and-shoot” approach

#### LODs, LOQs, linear range, and carryover

LODs, LLOQs, ULOQs, and carryover ratios for all compounds in both methods are given in Table S3. Table 1 summarizes the median LLOQs in solvent and plasma, as well as the ULOQs in solvent for all analytes grouped by analytical method, compound class, and derivatization state. Notably, compounds derivatized on two amino groups exhibited substantially lower median LLOQs in the derivatization method compared to the “dilute-and-shoot” method. In solvent, lower LLOQs were generally observed for the derivatization method across all derivatization states and compound classes, with the exception of biogenic aminess, where non-derivatized compounds yielded lower LLOQs in the “dilute-and-shoot” method. Consideration of the dilution factors of the concentrated plasma samples (1:30 in the derivatization method, 1:5 in the “dilute-and-shoot” method, *v:v*) minimized the gap between the two methods. Although the derivatization method still tended to result in lower LLOQs in plasma overall, the “dilute-and-shoot” method produced lower median LLOQs compared to the derivatization method for non-derivatized compounds. Regarding ULOQs, the “dilute-and-shoot” method consistently resulted in higher ULOQs across all compound classes and derivatization states.

**Table 1:** Summary of LLOQs and ULOQs of non-derivatized compounds and compounds derivatized at one (1 × deriv) or two (2 × deriv) functional groups in the derivatization method. Median values are shown with the respective minimum and maximum values in brackets. *AA* stands for amino acids, *AR* for amino acid-related compounds, and *BGA* for biogenic amines. The number of compounds is given in brackets after the derivatization state

Method	Compound Class	Derivatization State (No.)	LLOQ in solvent	LLOQ in plasma	ULOQ in solvent
Derivatization method	AA	1 × deriv (18)	8 (0.6–100)	200 (20–3000)	1000 (300–3000)
		2 × deriv (1)	10 (10–10)	300 (300–300)	300 (300–300)
	AR	1 × deriv (23)	3 (0.3–10)	90 (9–300)	1000 (30–3000)
		2 × deriv (1)	1 (1–1)	30 (30–30)	300 (300–300)
		Not deriv (14)	5 (1–300)	100 (30–9000)	650 (30–3000)
	BGA	1 × deriv (10)	2 (0.3–10)	50 (9–300)	800 (100–3000)
		2 × deriv (4)	20 (10–200)	600 (300–6000)	450 (300–3000)
		Not deriv (6)	2 (0.3–30)	50 (9–900)	100 (30–300)
“Dilute-and-shoot” method	AA	1 × deriv (17)	50 (20–2000)	300 (90–10,000)	10,000 (1000–25,000)
		2 × deriv (1)	400 (400–400)	2000 (2000–2000)	8000 (8000–8000)
	AR	1 × deriv (19)	9 (0.2–100)	50 (1–600)	1000 (100–25,000)
		2 × deriv (1)	200 (200–200)	1000 (1000–1000)	4700 (4700–4700)
		Not deriv (14)	7 (0.6–100)	40 (3–500)	6300 (24–50,000)
	BGA	1 × deriv (9)	3 (0.5–2000)	20 (2–10,000)	1000 (300–20,000)
		2 × deriv (3)	1000 (1000–1000)	5000 (5000–5000)	10,000 (10,000–25,000)
		Not deriv (6)	0.8 (0.5–20)	4 (3–90)	160 (22–1000)

The extent of the linear range, expressed as a ratio of ULOQ/LLOQ, was < 100 for 25 compounds in the derivatization method and for 29 compounds in the “dilute-and-shoot” approach. In the derivatization method, 37 compounds had a ratio between 100 and 1000, and 15 compounds exhibited an ULOQ/LLOQ ratio above 1000. Similarly, 31 compounds had a ratio between 100 and 1000 in the “dilute-and-shoot” method, and 10 compounds exceeded a ratio of 1000. Table 2 divides the ULOQ/LLOQ ratios by derivatization status. Compounds derivatized at two positions exhibited the narrowest linear range, whereas single derivatization resulted in the greatest proportion of metabolites with a linear range of at least three orders of magnitude.

**Table 2:** Number of compounds with an ULOQ/LLOQ ratio < 100, between 100 and 1000, and ≥ 1000 for non-derivatized compounds and compounds derivatized at one or two functional groups in the derivatization method and the same compounds in the “dilute-and-shoot” approach without derivatization

	ULOQ/LLOQ	Derivatization method	“Dilute-and-shoot” method [16]
Not derivatized compounds	< 100	10	8
	100–1000	8	5
	≥ 1000	2	7
1 × derivatized compounds	< 100	10	16
	100–1000	28	26
	≥ 1000	13	3
2 × derivatized compounds	< 100	5	5
	100–1000	1	0
	≥ 1000	0	0

In the case of amino acids for which dedicated internal standards were available, calibration curves constructed from peak area ratios (analyte/internal standard) showed a greater linear range than calibration curves established from peak areas (Table S3). As the internal standard was equally affected by detector saturation or non-linear effects during the ionization process, the linear range of analytes with dedicated internal standard was extended, in part even by one order of magnitude.

In general, the carryover rates were low in the derivatization method, with only seven compounds exceeding 1%, and six of them not being derivatized. Thyroxine was the only derivatized compound exceeding a carryover rate of 1%, and TMAO and *N*-methylalanine were the only compounds with carryover rates > 5%. In contrast, 32 compounds showed a carryover rate > 1% in the “dilute-and-shoot” method. Carryover rates > 5% were observed for the amino acids arginine, glutamic acid, and serine, the amino acid-related compounds betaine and ornithine, as well as for the biogenic amine putrescine. Hence, RP-HPLC-MS/MS analysis after derivatization with PITC showed lower carryover than analysis of native compounds by the HILIC “dilute-and-shoot” method.

#### Apparent recoveries

Apparent recoveries of individual metabolites in pig plasma after derivatization determined in concentrated (i.e., 1:30 diluted) and, for compounds exceeding the ULOQ in concentrated samples, additionally in 1:1500 diluted samples are given in Table S4. Table 3 summarizes the average RAs of the investigated compound classes, further stratified by derivatization status. In both tables, already published results from the “dilute-and-shoot” method are given for comparison.

**Table 3:** Average apparent recoveries (RAs) ± standard deviation of the investigated metabolites in concentrated and diluted pig plasma samples measured by the derivatization method and the “dilute-and-shoot” method

	Derivatization method				“Dilute-and-shoot” method [16]			
	1:30		1:1500		1:5		1:100	
	RA ± std dev	*n*	RA ± std dev	*n*	RA ± std dev	*n*	RA ± std dev	*n*
Amino acids	116 ± 26	19	141 ± 33	19	72 ± 14	18	83 ± 15	16
Amino acids plus IS	92 ± 9	19	95 ± 16	19				
Amino acid-related (not derivatized)	79 ± 33	12	104 ± 43	4	93 ± 15	14	93 ± 8	8
Amino acid-related (1 × derivatized*)	138 ± 31	23	177 ± 47	7	83 ± 14	19	83 ± 12	13
Biogenic amines (not derivatized)	58 ± 24	6			89 ± 11	6	91 ± 7	5
Biogenic amines (1 × derivatized*)	94 ± 19	10			74 ± 19	9	90 ± 10	2
Biogenic amines (2 × derivatized*)	109 ± 16	4			68 ± 6	3		

*derivatized refers to the number of derivatized functional groups per metabolite in the derivatization method

RAs of amino acids in concentrated samples were on average 116% (84–169%). In 1:1500 diluted samples, the average RA of amino acids was 141% (99–212%). Evaluation based on peak area ratios of the analytes and their dedicated internal standards improved the average RAs in concentrated and diluted samples to 92 and 95%, respectively, and also improved the repeatability. The benefit of internal standards added prior to sample preparation is that both losses during sample work-up and mass spectrometric matrix effects are compensated. However, for two compounds, the used internal standard mixture composed of deuterated amino acids (where hydrogen atoms are replaced by deuterium only in the “R” part of the amino acids) is not or only partially suited. As mentioned above, in the case of glycine, the first isotope peak of naturally occurring glycine disturbs the transition of the internal standard. If natural glycine concentrations are high, the peak area of the internal standard is increased, rendering internal standard correction unreliable. The second amino acid compromised by the internal standard mixture is histidine, which coelutes with an intense impurity from the internal standard mixture. To ensure accurate quantification of histidine, samples have to be analyzed without addition of internal standard to samples and calibration curves, or a different, e.g., ^13^C-labelled, internal standard mixture has to be chosen.

In both concentrated and diluted samples, RAs of singly derivatized amino acid-related compounds were mostly higher than 100%, indicating matrix enhancement. On the contrary, RAs of non-derivatized amino acid-related compounds were either close to 100% or reduced due to matrix suppression, resulting in an average RA of 79% for concentrated samples. Similarly, RAs of non-derivatized biogenic amines in concentrated samples were mostly affected by matrix suppression, resulting in an average RA of 58%. All non-derivatized amino acid-related compounds and biogenic amines that showed matrix suppression eluted very early in the chromatogram, with retention times < 1 min. The observed coelution of analytes, both with each other and likely with matrix components, is presumed to contribute to matrix-induced signal suppression. Average RAs of singly or doubly derivatized biogenic amines were satisfactory with 94 and 109%, which might be attributed to greater retention and better chromatographic separation of the derivatized compounds.

In general, RAs determined by the “dilute-and-shoot” method were closer to 100% than those obtained in the derivatization method. In addition, standard deviations were lower in the "dilute-and-shoot" method. The better RAs in the “dilute-and-shoot” method may result from more effective separation from matrix components - particularly for non-derivatized compounds - and the absence of impurities introduced by derivatization reagents.

#### Repeatability

The repeatability of the metabolites was similar in the derivatization and in the “dilute-and-shoot” method, with average values around 10% (Table S5). Especially in the derivatization method, the repeatability was better for analytes in concentrated samples compared to diluted samples. Consideration of the internal standards by evaluating peak area ratios (analyte/IS) improved the repeatability in concentrated samples. In diluted samples, peak areas of some internal standards were small, resulting in poorer repeatability.

### Application to plasma from different species

The concentrations of amino acids, amino acid-related compounds and biogenic amines in plasma from different species determined by the derivatization method and the “dilute-and-shoot” method are listed in Table S6. Table 4 presents a comparison of amino acid concentrations - limited to those with certified reference values in human NIST plasma - measured by both the derivatization method and the “dilute-and-shoot” approach. Overall, concentrations obtained by both methods matched the certified values. The higher value of arginine obtained by the “dilute-and-shoot” method might be due to correction by the RA (54%) that was determined for pig, but not for human plasma. Leucine concentrations were lower than the certified value in both methods. This might be due to differences in the reference standard. Another explanation might be that the quantifier transition for leucine is not 100% specific, as the fragment ion *m/z* 43 is also formed from the parent ion of isoleucine, albeit to a much smaller percentage, resulting in a slightly increased slope of the calibration curve if isoleucine is also present in the calibration solution.

**Table 4:** Concentrations of amino acids in the human NIST reference plasma sample determined by the derivatization and by the “dilute-and-shoot” method

	c in human NIST plasma (mg/L)			% of certified value	
	Certified values	Derivatization method	“Dilute-and-shoot” method [16]	Derivatization method	“Dilute-and-shoot” method
Alanine	26.7	26.9	22.4	100	84
Arginine	14.2	16.6	23.9	117	168
Isoleucine	7.27	8.07	6.82	111	94
Leucine	20.4	12.8	15.9	63	78
Lysine	20.4	22.8	23.6	112	116
Methionine	3.33	4.52	2.15	136	65
Phenylalanine	8.36	9.30	8.29	111	99
Proline	20.3	24.0	15.9	118	78
Serine	10.1	9.60	9.22	95	92
Threonine	14.2	14.6	11.9	102	84
Tyrosine	10.4	9.48	9.77	91	94
Valine	21.3	20.9	17.1	98	80

**Fig. 2:**
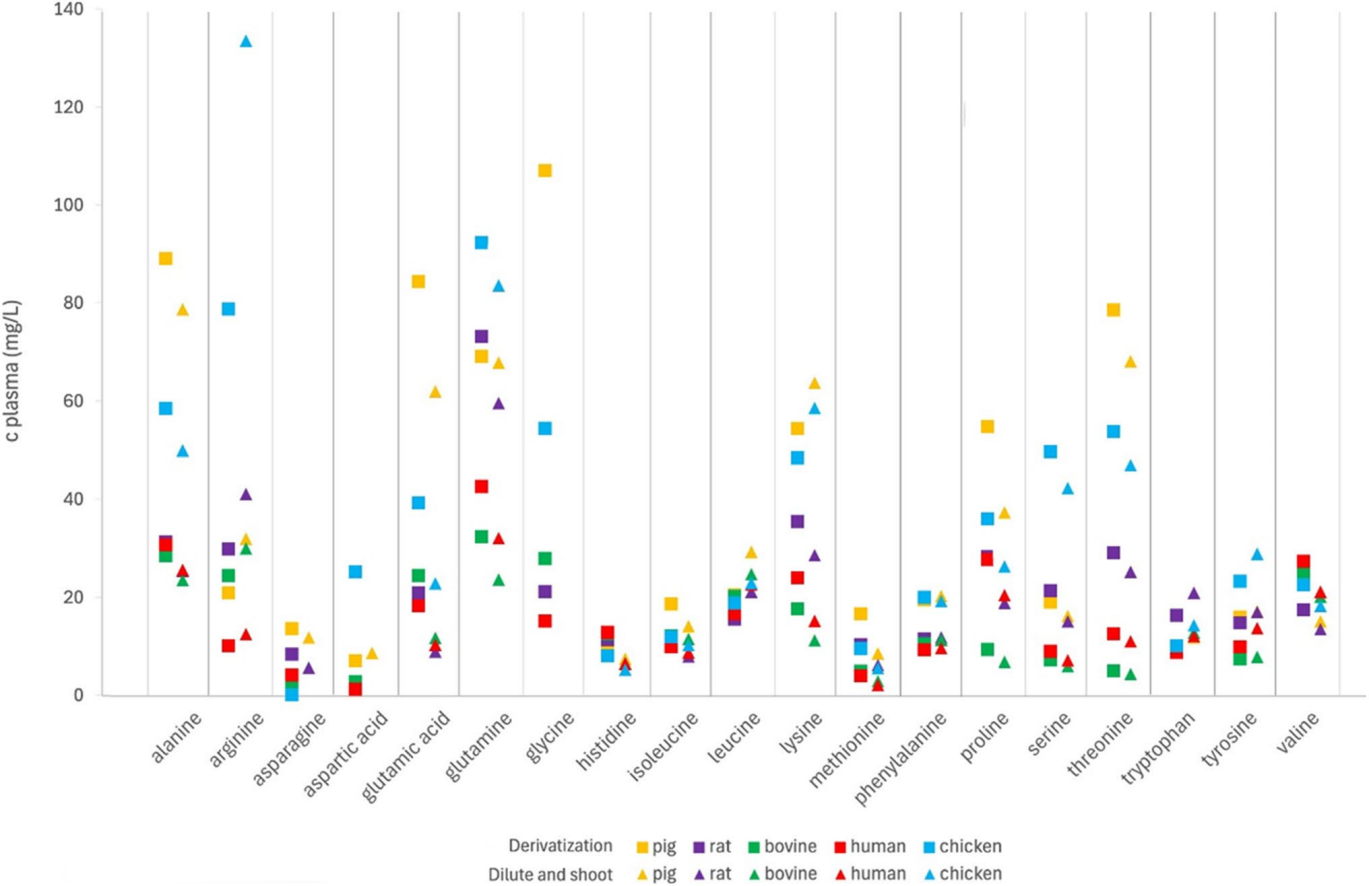
Comparison of concentrations of amino acids in plasma (mg/L) from different species determined by the derivatization method and the “dilute-and-shoot” method

A graphical comparison of the concentrations of amino acids obtained by the two methods is presented in Fig. 2. Generally, amino acid concentrations determined by the two approaches were similar, with some exceptions. For instance, concentrations of histidine determined by the derivatization method were higher by approximately a factor of 2. The reason for that is the disturbance of both quantifier and qualifier transition of histidine by an impurity from the internal standard mixture, which renders the histidine concentrations determined by the derivatization method unreliable unless samples are additionally analyzed without internal standard. In the case of glutamic acid, concentrations matched for pig plasma but differed by a factor of 2 for the other matrices, indicating different matrix effects of glutamic acid in the matrices that had not been validated. Due to high LLOQs in the “dilute-and-shoot” method and comparably low plasma concentrations, asparagine and aspartic acid were below the LLOQ in the “dilute-and-shoot” approach in all matrices but pig plasma and, in the case of asparagine, rat plasma. Glycine could not be reliably determined by the “dilute-and-shoot” approach as the only possible SRM transitions were non-specific and therefore more susceptible to matrix interference, leading to a gross overestimation of the measured values in the NIST sample [16].

**Fig. 3:**
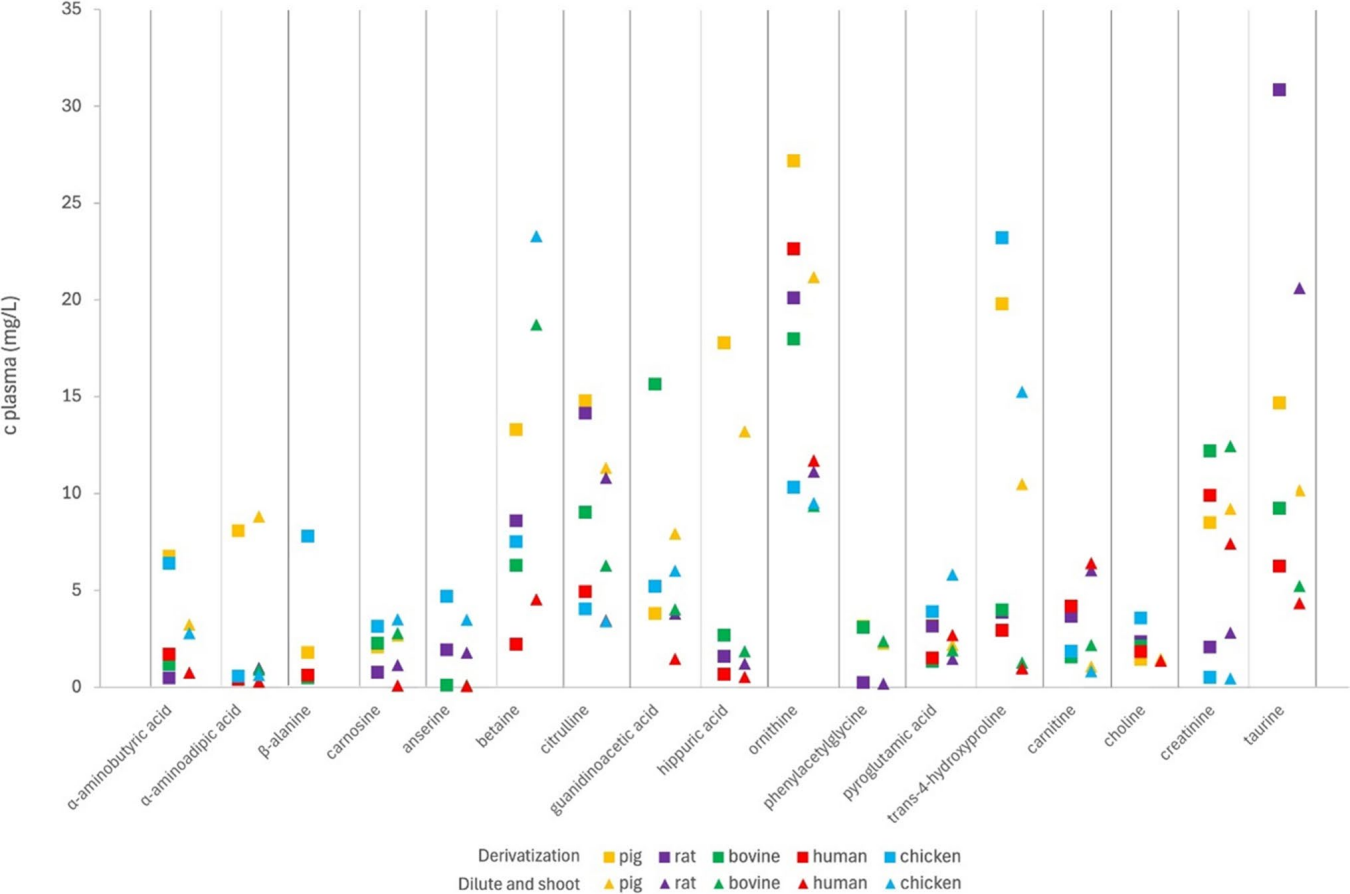
Comparison of concentrations of selected amino acid-related compounds and biogenic amines in plasma (mg/L) from different species determined by the derivatization method and the “dilute-and-shoot” method

A comparison of the concentrations of selected amino acid-related compounds and biogenic amines as determined by the two analytical methods is given in Fig. 3. Notably, for amino acid-related compounds and biogenic amines, discrepancies in concentrations were observed in both directions. These differences can be attributed, in part, to the use of distinct standard mixtures - despite both being prepared from the same stock solutions - as well as the extrapolation of validation data derived from pig plasma to the other matrices. In the following section, potential additional sources of variation in analyte concentrations between the two methods will be discussed for selected compounds. Additional notes to compounds not mentioned in the main article are provided in Table S6.

As mentioned above, the isomers AABA, BABA, and GABA are baseline-separated after derivatization, whereas *N*-methylalanine and choline coelute. Interestingly, chromatograms recorded by the derivatization method showed an additional unidentified peak at the SRM transition of BABA. According to Wang et al. [20], this compound might be β-aminoisobutyric acid (BAIBA), which occurs in a similar concentration range as GABA in human plasma. Due to high concentrations of choline in all investigated plasma samples, as well as due to the disturbance of the quantifier transition of *N*-methylalanine by the qualifier transition of choline and the very low intensity of the specific qualifier transition of *N*-methylalanine, *N*-methylalanine could not be determined by the derivatization method. In the “dilute-and-shoot” approach, GABA, BABA, and *N*-methylalanine were below the respective LLOQs, whereas AABA could be quantified in plasma of pigs, humans, and chickens. Due to the complexity of the separation problem, we had originally wrongly quantified BABA in our first paper [16], but comparison with the values from the derivatization method shed light on this error. ADMA and SDMA are well separated in the derivatization method, but show substantial coelution in the “dilute-and-shoot” method. However, in addition to an unspecific SRM transition, a specific SRM transition is also available for both compounds in both methods. Comparison of the concentrations obtained in the derivatization method with those published for the “dilute-and-shoot” approach [16] showed considerable differences. Close inspection of the original data obtained by the “dilute-and-shoot” method revealed that incorrectly, the non-specific transitions had been used as quantifier for ADMA and SDMA. Re-evaluation of the data using the specific SRM transition resulted in closely matching values for each metabolite. Another example of different isomer separation are 3-hydroxyproline, cis-4-hydroxyproline, and trans-4-hydroxyproline, which coelute in the “dilute-and-shoot” method. Despite early elution in the derivatization method, trans-4-hydroxyproline is baseline-separated from 3-hydroxyproline and cis-4-hydroxyproline, which partly coelute. The latter are below the LLOQ in the derivatization method, but trans-4-hydroxyproline can be determined. Interestingly, the derivatization method yields 2–3 times higher values than the “dilute-and-shoot” approach. Detailed examination of the chromatograms obtained by the “dilute-and-shoot” method revealed a later-eluting peak at the same SRM transition as hydroxyproline. This observation suggests that a coeluting compound, potentially also undergoing derivatization, may be present alongside trans-4-hydroxyproline. Notably, the chromatographic peak corresponding to trans-4-hydroxyproline exhibits a shoulder, as illustrated in Fig. 1, further supporting the possibility of coelution. The examples of the isomers BABA, ADMA, SDMA, and trans-4-hydroxyproline highlight the importance of cross-validating results obtained by one method with another, ideally an orthogonal method.

As derivatization resulted in lower LLOQs of derivatized compounds, some low-abundance amino acid-related compounds and biogenic amines were below or very close to the LLOQ in the “dilute-and-shoot” method. Examples are 5-aminovaleric acid, cadaverine, kynurenine, putrescine, and serotonin. Conversely, several non-derivatized compounds were not or only little retained in the derivatization method. In addition to worse LLOQs in plasma (due to the higher dilution factor), coelution with each other and matrix compounds and, in part, non-specific transitions resulted in values below the LLOQ or less reliable values in the derivatization method. This applied to 3-indoleacetic acid, 3-indolepropionic acid, betaine, tryptophan betaine, trimethylamine-*N*-oxide, and trigonelline.

In the cases of *N*-acetyltyrosine, stachydrine, guanidinoacetic acid, and pyrrolidine, that were not derivatized themselves, derivatization resulted in bad calibration curves which hindered reliable quantification. Calibration curves of spermine and spermidine, that were mainly derivatized twice, but to a small extent also three times (spermidine) and three and four times (spermine), also had mediocre correlation coefficients. *N*-methylalanine could not be detected at all after derivatization. We hypothesize that unexpected derivatization products that escaped detection might have formed to some extent.

Finally, pyroglutamic acid and creatine are examples of compounds that are partially derivatized. In the present work, we evaluated both the derivatized and the non-derivatized form. For pyroglutamic acid, results were very similar. In the case of creatine, values differed especially for human NIST reference plasma, human, and pig plasma. In all three matrices, the non-derivatized form yielded lower values than the “dilute-and-shoot” method, whereas the derivatized form gave rise to higher concentrations than those determined by the “dilute-and-shoot” approach. Averaging concentrations of derivatized and non-derivatized creatine yielded more similar values to those determined by the “dilute-and-shoot” method.

### Summary of the method comparison

Table 5 provides a summary of the advantages and limitations of both approaches for determination of amino acids, amino acid-related compounds, and biogenic amines in plasma samples. The derivatization method demonstrated significant advantages in terms of enhanced sensitivity for derivatized compounds, with lower LLOQs observed for most singly and doubly derivatized metabolites. This improvement was particularly notable for low-abundance compounds, such as certain amino acid-related compounds and biogenic amines, which were below the LLOQ in the “dilute-and-shoot” method. Additionally, derivatization improved chromatographic separation for several isomeric compounds, enabling more reliable quantification. However, the derivatization process introduced challenges, including matrix effects, coelution with impurities from the derivatization reagent and from the used internal standard mixture, and calibration issues for certain compounds, such as histidine, guanidinoacetic acid, and *N*-acetyltyrosine. Highly polar non-derivatized compounds, such as betaine and trimethylamine-*N*-oxide, were better quantified using the “dilute-and-shoot” method due to their poor retention in RP-HPLC compared to HILIC and higher LLOQs in the derivatization approach.

**Table 5:** Summary of key features of each method

	Derivatization method	“Dilute-and-shoot” method
Sample preparation	• Tedious • Many pipetting steps • Two evaporation steps • Error-prone • Limited stability of derivatized sarcosine	• Fast • Easy
Chromatographic separation	Better separation of • AABA and BABA • ADMA and SDMA • Alanine, β-alanine, sarcosine • 3-Hydroxyproline, cis-4-hydroxyproline, and trans-4-hydroxyproline • Spermidine	Better separation of • 1-methylhistidine and 3-methylhistidine • Valine and norvaline
LLOQs	• For compounds derivatized in the derivatization method, LLOQs were lower than in the “dilute-and-shoot” method in solvent solution and similar in plasma • Non-derivatized compounds had similar LLOQs in solvent solution in both methods, but LLOQs in plasma were lower in the “dilute-and-shoot” method	
Linearity	• Use of IS extends linear range • Highest linear range for singly derivatized compounds	
Carryover	• Derivatization reduced carryover rates	
Apparent recovery	• Matrix enhancement for most derivatized amino acids and amino acid-related compounds • Use of IS improves RAs • Matrix suppression for non-derivatized biogenic amines • Improved RAs for derivatized biogenic amines	• Less matrix effects • Improved RAs in diluted samples
Repeatability	• On average, similar repeatability as in the dilute-and-shoot method • Reduced repeatability in diluted samples • Greater danger of outliers	• Similar repeatability in concentrated and diluted samples
Undeterminable analytes	• *N*-Methylalanine • Norvaline	• β-Alanine, sarcosine (sum determination with alanine) • 3-Hydroxyproline, cis-4-hydroxyproline (sum determination with trans-4-hydroxyproline)
Unreliable analytes	• Histidine (if deuterated IS mixture is added) • Glycine if deuterated IS used • Bad calibration curves for *N*-acetyltyrosine, guanidinoacetic acid, stachydrine, spermine, spermidine, pyrrollidine	• Glycine
Troublesome metabolites during analysis of plasma samples	• Impure peak of trans-4-hydroxyproline • Several early eluting non-derivatized compounds < LLOQ or disturbed by matrix compounds (3-indoleacetic acid, 3-indolepropionic acid, betaine, tryptophan betaine, trimethylamine-*N*-oxide, trigonelline)	• Quantification of AABA, BABA, GABA, and *N*-methylalanine requires the evaluator’s unlimited attention • Several biogenic amines < LLOQ (5-aminovaleric acid, cadaverine, kynurenine, putrescin, serotonin)

Due to multiple pipetting and evaporation steps, the derivatization method is more error-prone than the “dilute-and-shoot” approach. Therefore, use of internal standards is highly recommended for the derivatization method - on the one hand to detect possible outliers, on the other hand for efficient correction of matrix effects. The “dilute-and-shoot” approach does not require internal standards, which saves money and time and avoids introduction of potential impurities from the IS mixture.

Finally, the choice of which method to use depends on several factors: Are there crucial analytes that cannot be determined in one of the methods? Which LLOQs are required to determine the analytes in question? How sensitive is the used mass spectrometer? How many samples are to be analyzed? We advise using the derivatization method if the focus is on derivatized compounds that benefit from enhanced ionization, lower LLOQs, improved chromatographic separation, and if carryover reduction is critical for the analysis. If the focus is on non-derivatized compounds (e.g., betaine, trimethylamine-*N*-oxide, trigonelline) that are better retained and quantified without derivatization, the “dilute-and-shoot” method is preferable. Likewise, we advise using the “dilute-and-shoot” approach if the study involves high-throughput applications of large sample cohorts, requires minimal sample handling, and if a simpler, faster, and less error-prone sample preparation process is preferred. In addition, if matrix effects and coelution with impurities are a concern, the “dilute-and-shoot” method is preferable as it generally avoids complications introduced by derivatization.

## Conclusion

In this article, we provide the first validation of an LC-MS/MS-based method for determination of amino acids, amino acid-related compounds, and biogenic amines after derivatization with PITC in plasma. A detailed comparison with a previously established “dilute-and-shoot” approach was also conducted, highlighting the strengths and limitations of each approach.

The comparison of metabolite concentrations in plasma across various species revealed good agreement between the two methods for most analytes, with some discrepancies attributed to varying matrix effects in plasma from different species, coeluting impurities, differences in LLOQs, and different calibration standard mixes. The comparison of results obtained by the derivatization method and the “dilute-and-shoot” approach also highlighted the importance of using different methods for cross-validation, particularly for isomeric compounds and compounds suffering from otherwise undetected coelution like trans-4-hydroxyproline.

The favored method ultimately depends on the specific requirements of the study. If highest sensitivity for derivatized compounds and improved separation of isomers are required, the derivatization method is recommended. However, for non-derivatized compounds, the “dilute-and-shoot” method is more suitable, which stands out due to its simpler workflows, lower LLOQs for non-derivatized compounds in plasma, and reduced error potential. Together, these methods provide a versatile toolkit for targeted metabolomics, enabling researchers to tailor their analytical strategies to the unique requirements of their studies. Complementary use of both methods as complementary approaches for cross-validation will provide the most robust results for comprehensive metabolomics studies.

## Supplementary Information

Below is the link to the electronic supplementary material.

Supplementary file1 (DOCX 259 KB)

Supplementary file2 (XLSX 88 KB)

## Data Availability

All data are included in the paper and its electronic supplementary material.
