# Supplementary material for: Comparative evaluation of phenyl isothiocyanate derivatization and “dilute-and-shoot” methods for HPLC–MS/MS-based targeted metabolomics analysis of amine-containing metabolites in plasma samples
Source: Anal Bioanal Chem. 2025 Aug 30;417(26):5859–72. doi: 10.1007/s00216-025-06079-5 (PMC12532724; doi:10.1007/s00216-025-06079-5)
Supplement: Supplementary file 1 — Supplementary file1 (DOCX 259 KB) [file 216_2025_6079_MOESM1_ESM.docx]

**Comparative evaluation of phenyl isothiocyanate derivatization and “dilute-and-shoot” methods for HPLC-MS/MS-based targeted metabolomics analysis of amine-containing metabolites in plasma samples**

Kangkang Xu^1,2,§^, Markus Aigensberger^1,2,§^, Franz Berthiller^1,2^, Heidi E. Schwartz-Zimmermann^1,2^,*

^1^ BOKU University, Institute of Bioanalytics and Agro-Metabolomics, Department of Agricultural Sciences, Konrad-Lorenz-Straße 20, 3430 Tulln, Austria

^2^ Christian Doppler Laboratory for Innovative Gut Health Concepts of Livestock, 1210 Vienna, 1210, Austria

^§^ shared first authors

Correspondence: [heidi.schwartz@boku.ac.at](mailto:heidi.schwartz@boku.ac.at)


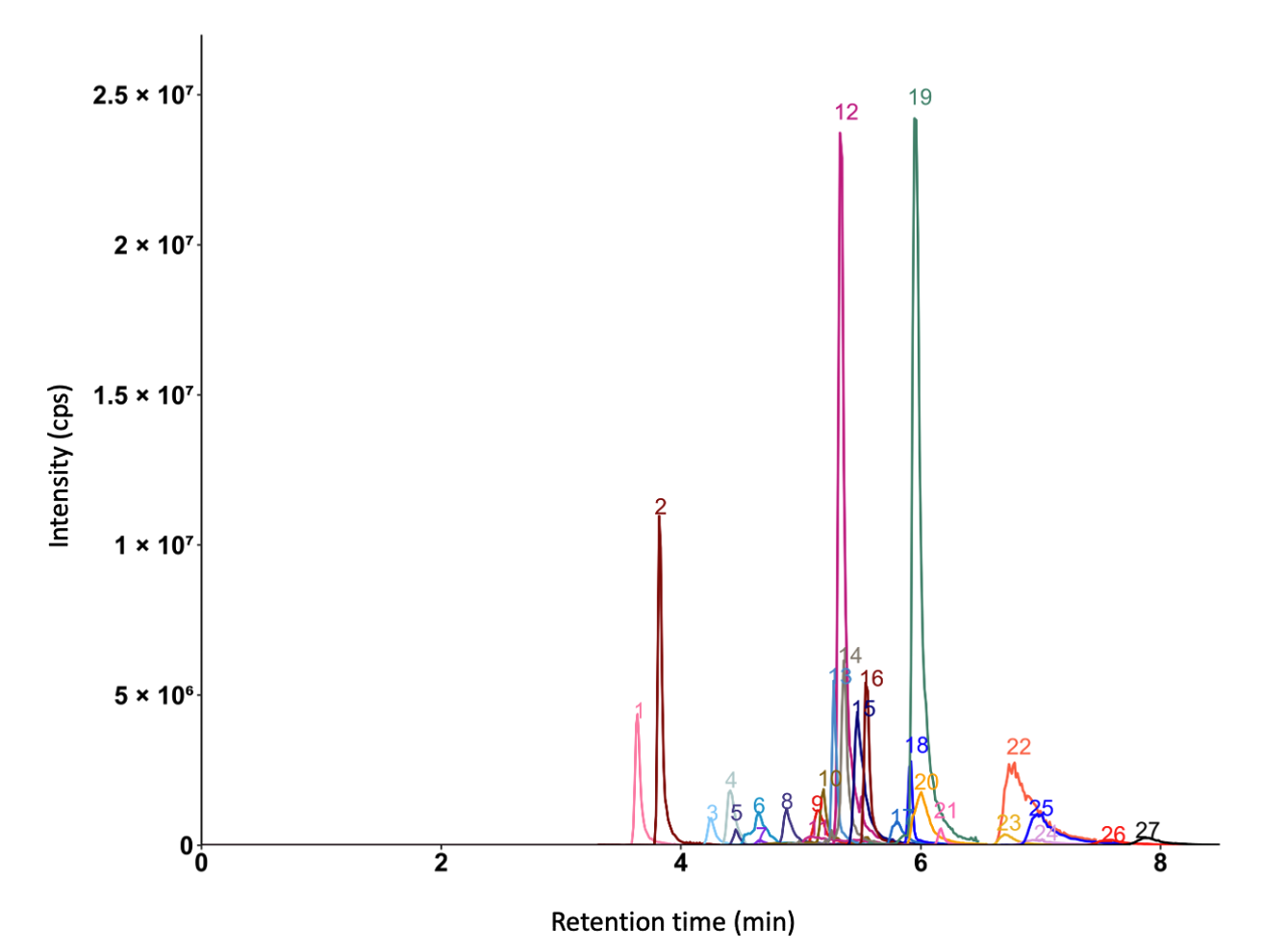


**Figure S1.** Extracted ion chromatogram of the QC plasma sample measured by HILIC-MS/MS. The peak intensity of betaine (peak 34) was scaled (divided by 3) for better visualization. 1: taurine; 2: creatinine; 3: tryptophan; 4: phenylalanine; 5: tyrosine; 6: leucine; 7: methionine; 8: valine; 9: threonine; 10: alanine; 11: glutamic acid; 12: proline; 13: trimethylamine-N-oxide; 14: choline; 15: glutamine; 16: creatine (non derivatized); 17: citrulline; 18: trigonelline; 19: betaine; 20: carnitine; 21: stachydrine; 22: arginine; 23: histamine; 24: ornithine; 25: lysine; 26: 3-methylhistidine; 27: 1-methylhistidine.

 
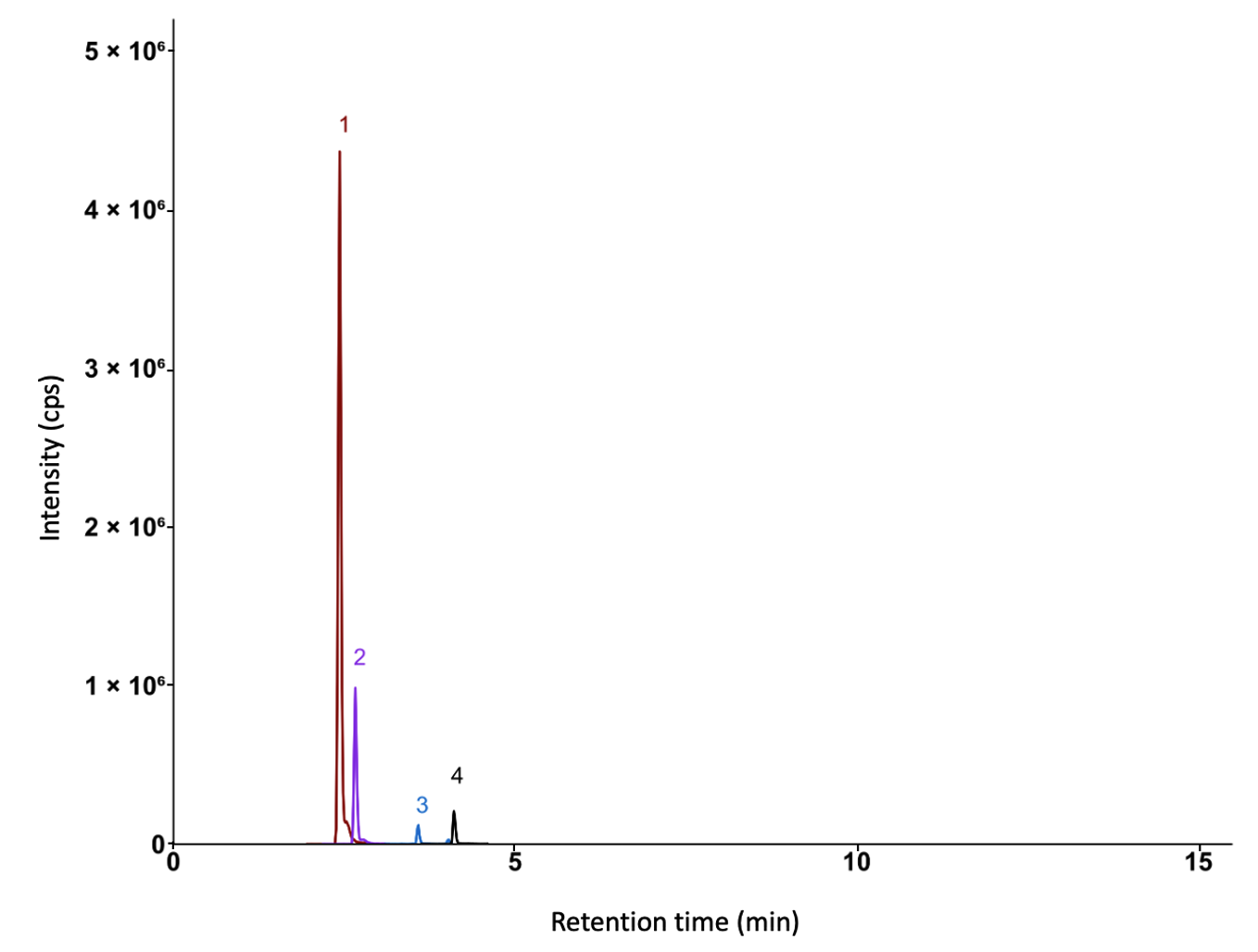


**Figure S2.** Extracted ion chromatogram of the QC plasma sample measured by RP-MS/MS. 1: hippuric acid; 2: phenylacetylglycine; 3: 3-indoleacetic acid; 4: 3-indolepropionic acid.
